# Recurrent Stroke Presentation in Neuro-Behcet Disease: Clinical Insights from a Case Report

**DOI:** 10.7759/cureus.104885

**Published:** 2026-03-09

**Authors:** Ali Al Janaahi, Samar i Mairajuddin, Raheel Muneer Ahmed Channa, Jai Perkash, Javeed A Dar, Abubaker Abdul Rahman Shaffi Al Madani

**Affiliations:** 1 Graduate Medical Education, Dubai Health, Dubai, ARE; 2 Neurology, Rashid Hospital, Dubai, ARE

**Keywords:** articles relevant to neurology and rheumatology, behcet syndrome, mri brain scan, neuro-behcet, recurrent ischemic stroke, stroke, young age stroke

## Abstract

Behcet disease is a form of multisystemic vasculitis, a rare and consequential manifestation of it, known as neuro-Behcet disease (NBD), associated with higher morbidity. We present a 32-year-old man with an ischemic stroke presentation as the initial sign of NBD. The presenting symptoms involved right-sided hemisensory loss associated with mild weakness, headache, dizziness, and gait imbalance, suggestive of a stroke. Initial computed tomography suggested a left thalamic infarction; subsequent magnetic resonance imaging revealed an irregular, ring-enhancing left thalamic lesion, raising concern for infectious or neoplastic causes. Extensive investigations ruled out infectious and neoplastic etiologies. Following the exclusion of other causes, the patient was managed with high-dose intravenous corticosteroids and then discharged on oral therapy for later outpatient follow-up. However, on discontinuing his medications, he presented with acute neurological symptoms indicating a recurrent ischemic stroke. This case highlights the importance of broad differential diagnosis in young patients presenting with stroke-like symptoms, as it can be a rare initial presentation of NBD. Furthermore, it also underscores the need for close follow-up and medication adherence in NBD patients, as relapse can lead to disabling and lifelong complications, as exemplified by our patient’s second stroke.

## Introduction

This article outlines the clinical course of a 32-year-old man presenting with complaints of hemisensory deficit, which was later confirmed as a case of neuro-Behcet disease (NBD). Behcet disease (BD) is a systemic vasculitis characterized by recurrent painful oral and genital ulceration, uveitis, and skin lesions [1].

In terms of distribution, the most common age group affected is 20-40 years, with equal incidence in both genders; however, the disease pattern of exacerbation and remission delays diagnosis, with studies showing it takes over five years to confirm the diagnosis [2].

The disease is associated with reduced quality of life due to its multidimensional, progressive impairment of physical, mental, and social functioning. As such, BD patients are at a higher risk of developing mental disorders and cognitive disturbances, with studies demonstrating that over 50% of BD patients develop depression and up to 60% develop anxiety disorders, with 20% of patients requiring a form of psychotropic medications [3].

The pathophysiology of the disease is complex and associated with exaggerated immune response, leading to hyperactivity in neutrophils and monocytes, along with pro-inflammatory cytokine release and vascular inflammation, which is associated with the increased risk of developing thrombosis and aneurysms. Genetic factors play a role, such as the strong association between HLA-B51 and the disease, along with newer genetic studies demonstrating that variation in the MICA gene region is associated with particular organ involvement of the disease, with polymorphisms in DDX60L being particularly linked with neurological involvement [3].

The most fatal phenotype of this disorder can be seen in NBD cases, and it is subdivided into parenchymal NBD involving areas of the cerebral hemispheres, brainstem, and spinal cord, or non-parenchymal NBD involving the vasculature, leading to intracranial aneurysm and cerebral venous thrombosis based on the involvement area seen on magnetic resonance imaging [4].

The most widely accepted diagnostic criteria for NBD were published in 2014 as the International Consensus Recommendation for NBD, which emphasized the need for combined clinical and symptomatic information, as well as investigations such as magnetic resonance imaging (MRI) screening and cerebrospinal fluid (CSF) laboratory findings, to confirm the diagnosis [5].

Using a combined approach for the diagnosis of NBD can facilitate the diagnosis process of the disease, as MRI alone may be easily confused with other neurological diseases, such as multiple sclerosis, with CSF analysis of interleukin-6 (IL-6) aiding in confirming the diagnosis of the disease; however, the elevation within CSF is not specific for NBD, indicating the importance of a multi-criteria diagnostic approach [4].

In terms of differences between the parenchymal and non-parenchymal NBD, parenchymal involvement is more common, accounting for around 75% to 80% of all NBD cases showing signs of meningoencephalitis, with the majority of cases involving the brainstem with potential involvement of the thalamus, basal ganglia, white matter, and spinal cord, with the potential of demonstrating pyramidal signs that appear bilaterally. The disease follows a relapsing-remitting pattern, with MRI usually demonstrating lesions in the upper brainstem extending into the thalamus and basal ganglia on one side [4].

Parenchymal NBD may also be divided into acute and chronic forms, with a meta-analysis demonstrating that acute cases are more commonly associated with fever and elevated CSF cell count. On the other hand, chronic cases were more associated with confusion, dementia, dysarthria, ataxia, brainstem atrophy, and abnormal cerebellar findings on MRI Brain. There is a potential association between smoking history and previous use of cyclosporine and the type of parenchymal NBD; however, due to this being found in a single study, appropriate correlation cannot be determined. Otherwise, the meta-analysis showed no difference in human leukocyte antigen-B51 status, age of onset, gender, any brainstem findings, abnormal thalamus findings, white matter involvement, basal ganglia involvement, cerebrospinal fluid (CSF) IL-6 levels, and CSF protein levels between the two subtypes [6].

On the other hand, non-parenchymal NBD usually occurs secondary to vascular complications, including cerebral venous thrombosis, aneurysm, dissection, and intracranial hypertension; unlike parenchymal NBD, this form follows a monophasic course, but recurrence may occur. In terms of imaging, venography would commonly display cerebral venous sinus thrombosis; however, it may rarely occlude the dural sinuses and cause venous infarcts [4].

While it is well known that BD involves neurological symptoms, an initial presentation of stroke is rare. We report a case of a young man presenting to the hospital with signs of stroke as an initial presentation of NBD, with a second recurrent stroke-like presentation on a different visit, highlighting the diagnostic and management challenges involved with the disease.

## Case presentation

A 32-year-old man, Algerian National, presented to the emergency department (ED) with a one-day history of sudden-onset weakness and sensory loss over the right side of his body. One week before this sudden onset of weakness and numbness, the patient experienced numbness on the right side of his face, which gradually spread downward over the following days and was noted to differ in sensation from the acute sensory loss he presented with. During his hospital presentation, the patient had associated symptoms of frontal headache, dizziness, nausea, and gait imbalance.

One week prior to the onset of initial neurological symptoms, the patient had developed fever and flu-like symptoms. The patient denied the presence of any previous similar episodes of neurological deficits, traumas, seizures, visual disturbances, or other neurological disorders. The patient’s family history was significant for diabetes with no other known conditions. The patient was a heavy smoker with no other significant medical history.

On examination, the patient was oriented, with a Glasgow Coma Scale (GCS) of 15/15 and intact higher mental functions. Abnormalities noted include ataxic gait, mild right-sided pronator drift, right-sided hypoesthesia, 4+/5 right upper limb strength, 4+/5 right lower limb strength distally, right-sided upgoing plantar reflex, right-sided dysmetria, and dysdiadochokinesia on the right. Muscle strength was graded based on the Medical Research Council (MRC) scale.

**Figure 1:**
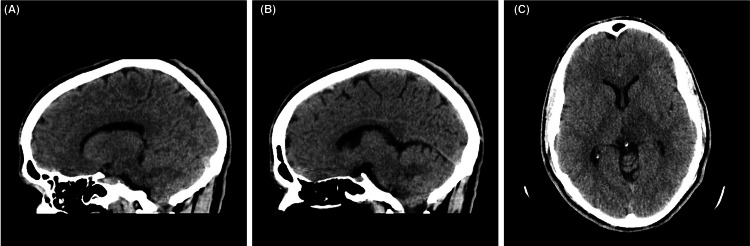
CT scan images (A) and (B) demonstrate a sagittal axis view, while image (C) demonstrates an axial axis view. This figure demonstrates the presence of a left thalamic lesion with involvement of the internal capsule’s posterior limb.

Labs showed an abnormal lipid profile with elevated triglycerides and low high-density lipoprotein, and complete blood count demonstrated elevated white blood cell counts, mainly neutrophils and monocytes. His National Institutes of Health Stroke Scale score was 4; however, computed tomography (CT) showed an ill-defined hypodensity in the left thalamus and the posterior limb of the internal capsule, as seen in Figure 1.

The patient was admitted to the high-dependency unit with constant monitoring and was loaded on aspirin 300 mg, clopidogrel 300 mg, and high-intensity atorvastatin 40 mg. Investigations for the young stroke workup were negative for any abnormalities. Trans-thoracic echocardiography showed no abnormal findings. Physiotherapy was provided to the patient during his entire stay.

**Figure 2:**
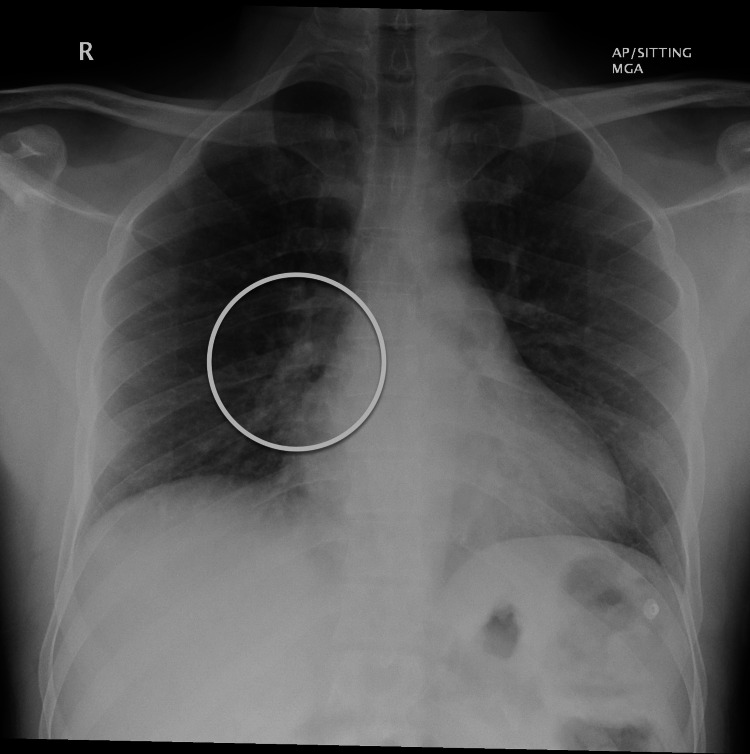
Presence of ill-defined opacities observed in the right pericardiac region, with the remaining lung field appearing normal and normal cardiac size.

**Figure 3:**
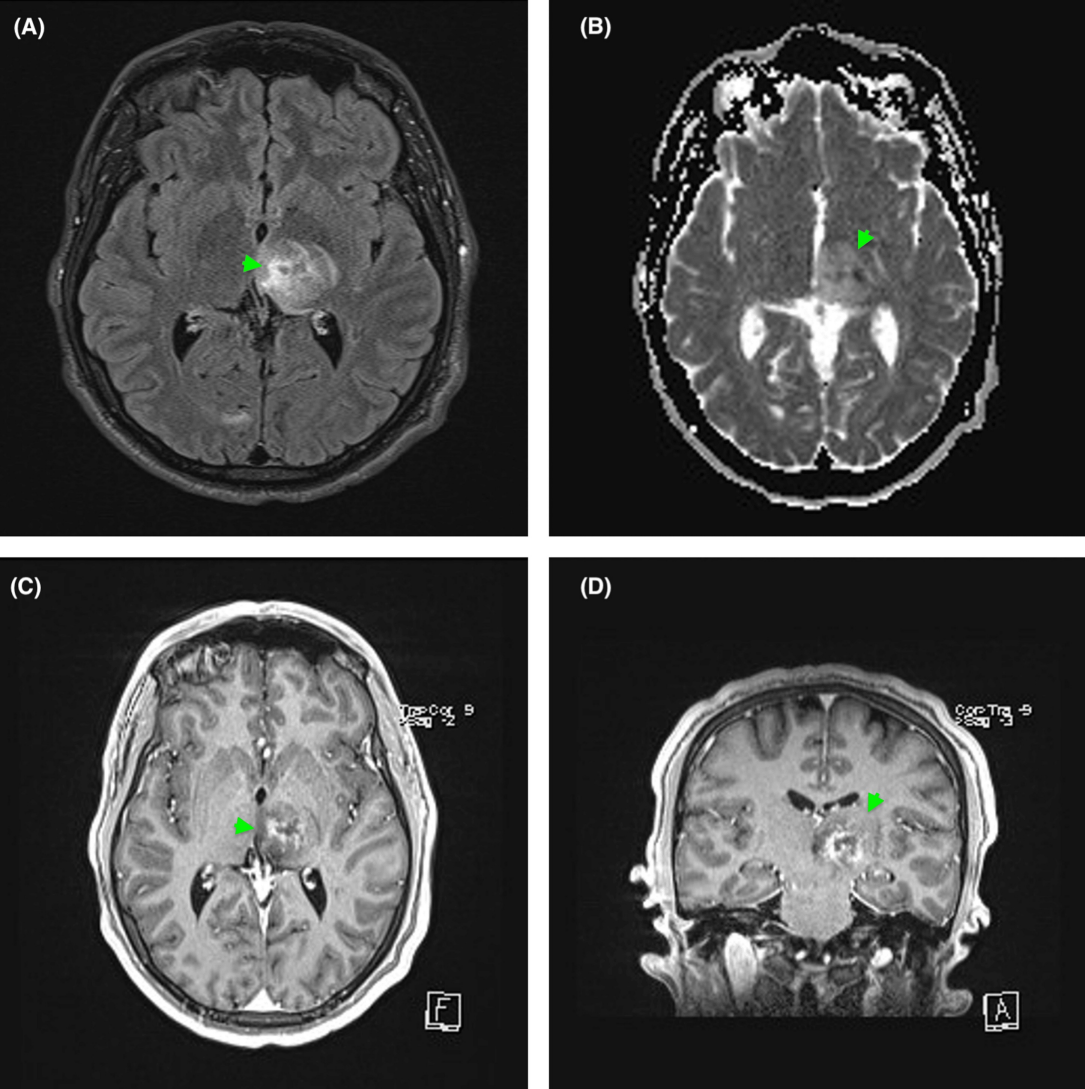
(A) Demonstrates an axial fluid-attenuated inversion recovery (FLAIR) post-contrast sequence. (B) Demonstrates an axial apparent diffusion coefficient (ADC) map sequence. (C) Demonstrates an axial T1-weighted post-contrast sequence. (D) Demonstrates a coronal T1-weighted post-contrast sequence. The brain MRI demonstrates a left-sided thalamic irregular ring-enhancing space-occupying lesion (SOL) associated with diffusion restriction. Images demonstrate the presence of a lesion surrounded by peri-focal oedema with a small, predominantly linear abnormal signal in the right inferior midbrain.

Chest x-ray demonstrated ill-defined opacities, as shown in Figure 2, and respiratory viral swab cultures and blood cultures were sent to rule out any potential source of infectious thromboembolic agents or other systemic infections. Respiratory culture was positive for *Haemophilus influenzae*. Given the presence of ring-enhancing thalamic lesions on MRI and noted opacities on X-ray, central nervous system (CNS) infection, such as tuberculosis, was considered; therefore, tuberculosis polymerase chain reaction (TB PCR) and acid-fast bacilli (AFB) culture were obtained. TB PCR, AFB smear, and culture were all negative. MRI of the brain demonstrated a left thalamic irregular, ring-enhancing, space-occupying lesion (SOL) with diffusion restriction. It was surrounded by perifocal oedema, along with a tiny, predominantly linear abnormal signal in the right inferior midbrain, suggestive of an incidental developmental venous anomaly, as seen in Figure 3.

As such, the patient was sent for a sexually transmitted disease workup, an immunity status workup, a lumbar puncture (LP) with cerebrospinal fluid (CSF) analysis, a paraneoplastic workup, and a tuberculosis (TB) workup. Due to the patient's history, differential diagnoses included atypical acute disseminated encephalomyelitis, CNS lymphoma, and NBD. The patient was transferred to the infection isolation unit with airborne precautions.

Pan-CT with contrast was performed to rule out any underlying pathology, such as disseminated TB and lymphoma, before initiating further management options, with the images demonstrating no thoracic or abdominal disseminated TB, malignancy, or pathological lymphadenopathy. As such, aspirin, clopidogrel, and atorvastatin were discontinued, with planned intravenous methylprednisolone (IVMP) 1 g daily for five days and intravenous (IV) pantoprazole. After the exclusion of disseminated malignancy or infection and the likelihood of NBD, IVMP was initiated, as high-dose corticosteroids are the first-line therapy for acute NBD.

On the day following medication administration, the patient reported significant improvement in numbness, with the ability to mobilize out of bed and tolerate oral feeding. Pneumonia panel initially returned positive for methicillin-resistant *Staphylococcus aureus*, *Streptococcus agalactiae*, and *Haemophilus influenzae*; suspected *H. influenzae* was repeated for confirmation. Pulmonology was consulted for appropriate management; however, as the patient was asymptomatic and afebrile with low-risk septic markers, immediate intervention was not needed, and it was recommended to wait for new panel results and TB testing to come out.

After the patient completed his five-day course of pulse methylprednisolone, he was started on prednisolone 60 mg once daily (OD) the following day and continued tapering dose. All repeat confirmatory infectious tests, TB workup, and CNS infection tests were negative, and airborne precautions were discontinued.

The patient was discharged in a stable condition with prednisolone 60 mg OD for 1 month, with consideration of starting a tapering dose in the outpatient visit. He was referred to an outpatient ophthalmology visit to rule out uveitis and was educated on the dangers of suddenly stopping steroids, with long-term potential side effects of his steroid medication.

On follow-up in the clinic, the patient demonstrated improvement on prednisolone with complete recovery of muscle power; however, persistent right-sided hemi-hypoesthesia and mild dysmetria were noted. Follow-up MRI of the brain and magnetic resonance spectroscopy (MRS) were to be performed in an outpatient setting, and prednisolone was decreased to 40 mg for two weeks, then to 20 mg for two weeks, and finally continued at 10 mg prednisolone in his follow-up visit after three months.

On his second follow-up, the patient reported improvement of symptoms with planned continuation of the previously agreed-upon management plan; however, two months after this visit, the patient presented to the ED with complaints of right-sided continuous headache, which started two days prior.

The patient stopped his medication suddenly prior to these episodes, as they were not available, and was seen by the neurology team in the ED. The patient was stabilized through symptomatic management. The patient was re-educated on the importance of following up on his medications and discharged.

Three days later, the patient presented again to the ED with complaints of generalized body weakness and inability to walk for the past two days following his discharge. On examination, he was noted to have facial asymmetry, severe upper and lower ataxia, and an inability to stand on his feet.

Muscle strength was graded based on the MRC scale. Left upper limb strength was 4/5 with weakened hand grip, left upper limb showed pronator drift, right-sided lower limb strength of 4+/5 with left lower limb strength of 3/5, down/left withdrawal on plantar response, bilateral finger to nose test positive, deep tendon reflex 2+, and knee reflexes were brisk. The left finger-to-nose test was impaired, with a tendency to fall when attempting to sit, an inability to lift the foot while standing, and leftward tilting.

Due to severe brainstem signs, ataxia, and facial palsy with marked deterioration in neurological status, the stroke protocol was initiated, with the conduction of a CT brain scan. GCS was 15/15, and the patient was fully oriented during his presentation.

The patient was readmitted with a second neurological event consistent with relapse of NBD. CT brain on new admission showed new lesions in the right thalamic/midbrain region, with a seemingly regressing left-side lesion compared to the previous CT.

**Figure 4:**
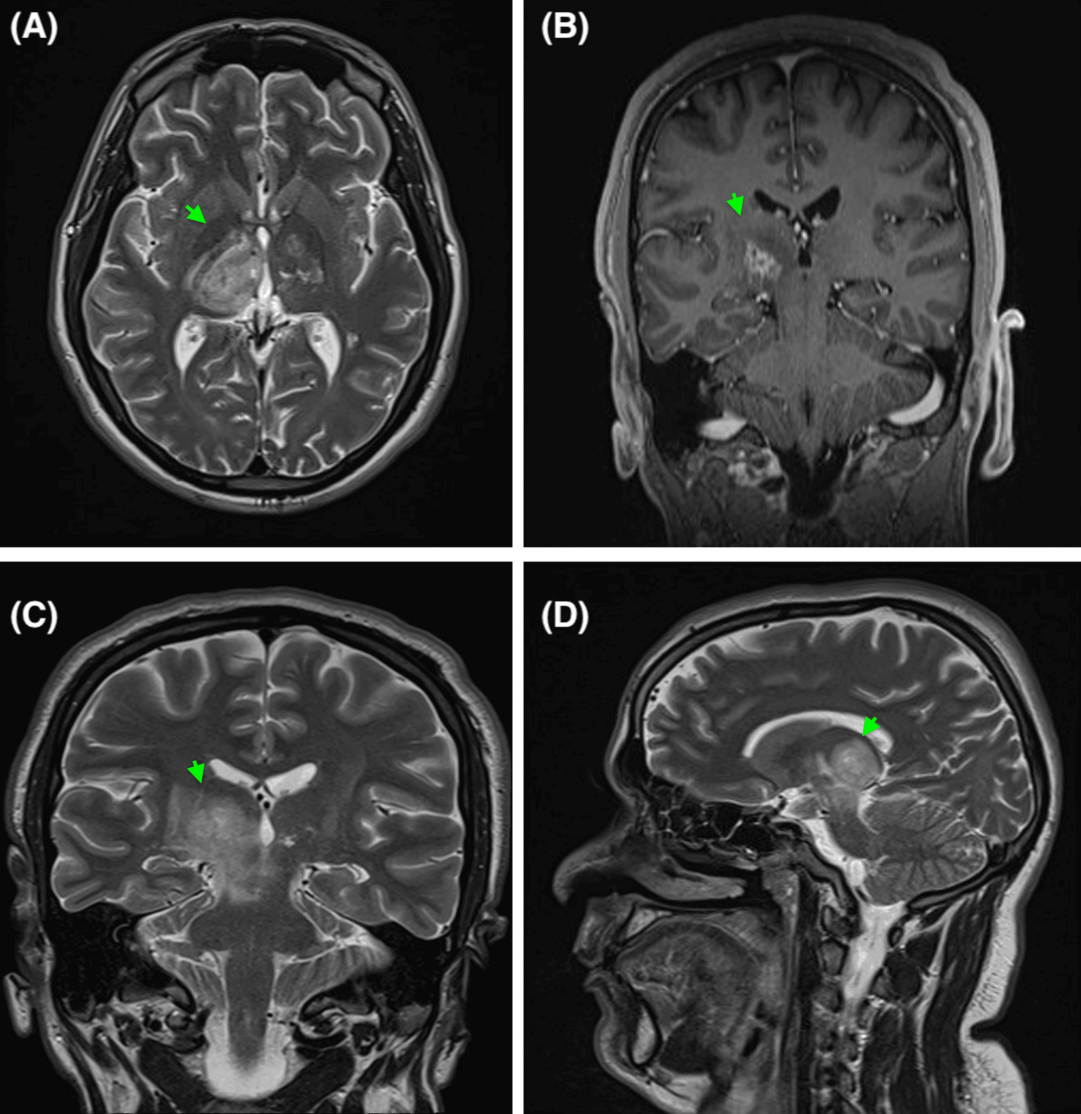
(A) Demonstrates an axial T2-weighted turbo spin echo (TSE) sequence. (B) Demonstrates a coronal T1-weighted 3D magnetization prepared rapid gradient echo (MPRAGE) post-contrast sequence. (C) Demonstrates a coronal T2-weighted TSE sequence. (D) Demonstrates a sagittal T2-weighted TSE sequence. The brain MRI demonstrates signs of significant interval regression of a previous left thalamic lesion alongside a newly developed right-sided thalamic heterogeneous enhancing lesion, and a proper aspect of the midbrain indicating disease progression.

MRI brain, compared with the previous one, showed significant interval regression of the previously seen irregularly enhancing left thalamic lesion, with a newly developed heterogeneous enhancing lesion in the right thalamus and the proper aspect of the midbrain, suggestive of a progressive course of the disease as seen in Figure 4. MRI thoracic and cervical spine with contrast showed no evidence of demyelinating plaques. Other repeat studies and imaging showed no significant findings.

MRS was performed, demonstrating significant interval regression of the irregularly enhancing left thalamic lesion, with newly developed heterogeneous enhancing lesions in the right thalamus and the right aspect of the midbrain. Lumbar puncture and CSF analysis demonstrated small to medium-sized lymphocytes, neutrophils, rare eosinophils, and macrophages with no signs of parasitic, fungal hyphae, or malignant cells being present.

The patient was provided with IVMP 1 g daily for five days, with consideration of starting disease-modifying drug/azathioprine. Once started on IVMP, the patient improved. No infection was noted on CSF investigation; however, it demonstrated an elevation in the CSF protein levels. Autoimmune panel results were negative for any potential mimickers of the disease.

The patient's case was consulted with the rheumatology team due to the patient not exhibiting any characteristic systemic features of the disease, such as recurrent oral/genital ulcers, acneiform nodules, a history of thrombosis, joint or eye complaints. The patient was planned for a brain biopsy and pathergy test; however, due to a lack of follow-up by the patient after discharge, the tests could not be performed. Prior to discharge, ophthalmological review was conducted with no significant findings being noted.

Once the patient was fully stabilized, discharge was planned on oral prednisolone 60 mg, tapering by 10 mg every 10 days to 10 mg; Escitalopram 10 mg OD; Azathioprine 25 mg twice daily; folic acid 5 mg OD; vitamin D 10,000 IU OD; and pantoprazole.

As such, due to the exclusion of other potential diagnosis and other causes of vasculitis such as primary CNS vasculitis being inconsistent with the patient’s presentation, the patient's age demographic and being from the North African region demonstrating the Silk Road distribution, the MRI and CSF findings indicative of NBD with characteristic neurological syndrome, and the patient's rapid clinical improvement in response to high-dose steroids along with the patients condition relapsing following the discontinuation of the medications all support the diagnosis of NBD.

## Discussion

The reported frequency of NBD is highly variable, with studies reporting rates ranging from 1.3% to 59%, which can be explained by multifactorial factors, leading to potential bias from geographical and ethnic variation, study design, and varying management protocols. In terms of geographical distribution, the greatest number of disease incidents are known to occur in the Silk Road network regions, including the Middle East, China, the Mediterranean, Japan, and Southeast Asia [7,8].

The prevalence of the disorder varies from country to country; for example, Turkey has the highest prevalence with an estimated range of 80-420 cases per 100,000 individuals based on the region; on the other hand, western countries have the lowest prevalence of the disease such as the United States with estimated range of 0.11 to 5.2 cases per 100,000 individuals [3].

The disease usually involves middle-aged individuals between 20 and 40 years, with studies demonstrating that men are more likely to develop NBD, with men having a greater risk for morbidity and mortality outcomes, not only in NBD but in BD in general. It is also important to note that due to the age range involvement, individuals older than 50 years old should have the more common disorders excluded prior to confirming the diagnosis, particularly strokes and non-specific changes in white matter on cranial MRI [7].

In terms of the development of neurological disorders in BD, it took approximately three to six years following the onset of systemic features to develop neurological disorders, with the incidence of neurological complications accounting for 9.3% of BD cases. Out of those neurological components, 75% had complications of meningoencephalitis, while 18% had vascular-related complications [7,8].

While neurological involvement is a known complication of BD, there is limited information regarding strokes being the initial presentation of the case, making the information highly valuable [9].

Key emphasis needs to be placed on the screening of patients for mental health-related disorders associated with BD in general, such as anxiety or depression, due to how, even though they have a high prevalence, they are underrecognized. Routine standardized screening through tools such as the Hospital Anxiety and Depression Scale or Beck Depression Inventory would support early intervention. Such measures are important due to the higher prevalence of suicidal ideation and suicidal attempts within this disease population [3].

In terms of NBD, clinical features may include signs of brainstem involvement such as ataxia, ophthalmoparesis, and pyramidal or sensory symptoms if lesions occur in the midbrain, and potential symptoms of dysarthria, dysphagia, and long tract signs if lesions involve lower brainstem structures. In terms of cerebral hemisphere involvement, the patient would present with signs of encephalopathy with focal neurological signs based on the site involved, with symptoms such as dysmnesia, dysphasia, and visual field defects [8].

Around 40% to 60% of BD patients develop significant disruption to their occupations, leading to work disability or early retirement, with it being more prominent in cases of neurological or ocular involvement, causing a significant socioeconomic burden. As such, patients would require appropriate vocational rehabilitation and social support to manage their disorder appropriately [3].

Other potential features include tumefactive lesions in the diencephalon, which can simulate primary or secondary tumors, isolated cranial neuropathy, optic neuropathy, audio-vestibular complications, chorea, oculopalatal tremor, dystonic tremor, and spinal cord involvement with lesions in the cervical and dorsal areas [8].

To diagnose BD, an individual should have recurrent ulcerations with at least three episodes of oral aphthous or herpetiform ulceration within a period of 12 months; furthermore, the patient should have at least two of the following symptoms including recurrent genital ulceration, eye lesions, skin lesions, or positive pathergy test based on the International Study Group (ISG) criteria for the diagnosis of BD in 1990 [10].

Traditionally, to diagnose NBD, the ISG criteria for BD should be met following the presence of clinical neurological syndromes, with objective neurological signs being observed within clinical settings. In addition to these clinical bases, at least one investigation showing abnormalities should be present, whether on neuroimaging or CSF analysis, that supports these findings. An example of a potential CSF finding supporting the diagnosis is elevated interleukin 6 (IL-6) [4]. However, it is important to note that in 2014, the diagnostic criteria for NBD were updated, allowing for the diagnosis of the disorder as probable NBD when the neurological presentation and neurological investigation findings, such as MRI and CSF, are highly indicative of the disease, even if the patient's presentation is not enough to fulfill the complete previous ISG diagnosis threshold that was previously established [5].

In terms of management, for cases of acute NBD, CSF analysis has to be performed to observe the cell count. If the cell count is not greater than 6.2/mm^3^, then other CNS disorders should be investigated. However, if it is elevated, then any infectious disease needs to be ruled out to start treatment on moderate to high-dose steroids with discontinuation of cyclosporine when administered [11].

If initial treatment with moderate to high-dose corticosteroids is insufficient, the patient may be initiated on corticosteroid pulse therapy or infliximab; however, if it is effective, the steroid should be tapered with the addition of colchicine. Then CSF analysis should be performed to measure IL-6, and if less than 17 pg/mL, then the patient should be followed up later on in an outpatient setting; however, if the target IL-6 level is not met, then the patient should be treated as a case of chronic NBD [11].

For chronic NBD cases, first, CSF analysis should be performed to measure IL-6 levels. If it is <17 pg/mL, then repeat brain MRI with focus on brainstem visualization for atrophy should be done, with a follow-up repeat CSF IL-6 measurement to be performed within two weeks. The findings of brainstem atrophy or IL-6 >17 pg/mL indicate a need for treatment with methotrexate, up to 16 mg/week. Following this treatment course, IL-6 levels need to be checked again and if within the target of <17 pg/mL, then methotrexate needs to be continued. However, if the target is not met, then infliximab should be added with an analysis to be performed again later on to measure IL-6. If the target level of IL-6 is not met, other biologic therapies should be considered; however, if the IL-6 levels normalize, infliximab should be continued [11].

It is also important to note that, in non-parenchymal NBD presenting with cerebral venous sinus thrombosis, standard anticoagulation may be provided for three to six months to manage the venous thrombus, alongside corticosteroids in the acute or subacute phases, for proper disease management [4].

## Conclusions

This case demonstrates the challenges involved in diagnosing and managing NBD, especially when it presents with uncommon initial features such as multiple ischemic strokes, emphasizing the importance of a detailed history for young stroke patients, providing multidisciplinary care, and considering BD in young individuals with no conventional risk factors of ischemic stroke.
